# Structural and biophysical characterization of the type VII collagen vWFA2 subdomain leads to identification of two binding sites

**DOI:** 10.1002/2211-5463.12807

**Published:** 2020-03-14

**Authors:** Jan M. Gebauer, Florian Flachsenberg, Cordula Windler, Barbara Richer, Ulrich Baumann, Karsten Seeger

**Affiliations:** ^1^ Institute of Biochemistry University of Cologne Germany; ^2^ Institute of Chemistry and Metabolomics University of Lübeck Germany; ^3^ Present address: ZBH – Center for Bioinformatics Universität Hamburg Hamburg Germany

**Keywords:** epidermolysis bullosa acquisita, integrin binding, laminin‐332, structure, type VII collagen, vWFA2

## Abstract

Type VII collagen is an extracellular matrix protein, which is important for skin stability; however, detailed information at the molecular level is scarce. The second vWFA (von Willebrand factor type A) domain of type VII collagen mediates important interactions, and immunization of mice induces skin blistering in certain strains. To understand vWFA2 function and the pathophysiological mechanisms leading to skin blistering, we structurally characterized this domain by X‐ray crystallography and NMR spectroscopy. Cell adhesion assays identified two new interactions: one with β1 integrin via its RGD motif and one with laminin‐332. The latter interaction was confirmed by surface plasmon resonance with a *K*_D_ of about 1 mm. These data show that vWFA2 has additional functions in the extracellular matrix besides interacting with type I collagen.

## Graphical abstract

Type VII collagen is an extracellular matrix protein, which is important for skin stability. The structure of the vWFA2 subdomain of type VII collagen has been determined, and it has been shown that vWFA2 interacts with integrins. In addition, a new interaction site for laminin‐332 was identified and characterized. These data provide new insights into vWFA2 functions in the extracellular matrix.

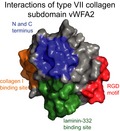

**Abbreviations**
EBA: epidermolysis bullosa acquisita
NH‐RDC: RDCs of backbone amides
RDC: residual dipolar couplings
SPR: surface plasmon resonance

Anchoring fibrils contain type VII collagen as major component and bind skin proteins belonging to the basal lamina and the underlying connective tissue. Anchoring fibrils are important in linking these skin layers together 1, 2. The central collagenous domain of type VII collagen has an N‐terminal cysteine knot 3 and shows several interruptions (Fig. 1). The longest interruption has been termed a hinge region 4 and is structurally disordered 5. The collagen triple helix is flanked by a large N‐terminal (NC1) and a small C‐terminal (NC2) noncollagenous domain 6. The NC1 domain consists of eleven subdomains and interacts with other extracellular proteins like laminin‐332, type I and IV collagen 7, 8, 9. The interaction with type I collagen is mediated by a subdomain termed vWFA2 that has homology to the von Willebrand factor A domain 3 10, 11.

**Figure 1:**
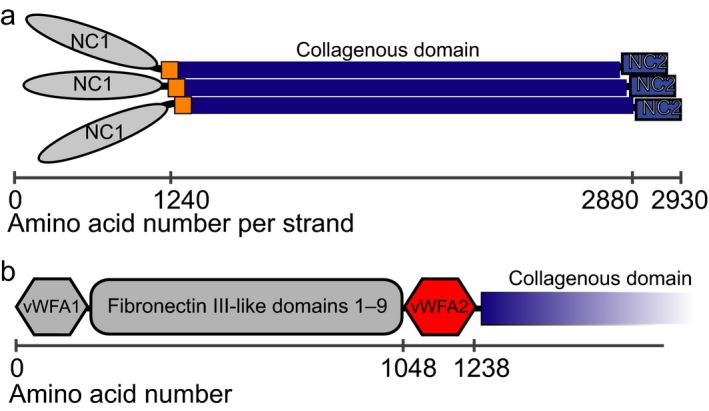
Domain architecture of type VII collagen. Type VII collagen forms homotrimers that later assemble to anchoring fibrils. Type VII collagen has a central collagenous domain (blue) with an N‐terminal cysteine knot (yellow). N‐ and C‐terminal are two noncollagenous domains NC1 and NC2 (A). NC1 subdomains have homology to adhesion domains and mediate important interactions of type VII collagen. The vWFA2 subdomain is N‐terminal of the triple‐helical region of type VII collagen (B). The amino acid sequence of the investigated protein is given in Fig. S1.

The importance of type VII collagen and its interactions with other skin proteins is highlighted by the skin blistering disease epidermolysis bullosa (EB) existing as a hereditary and an acquired form. Hereditary EB is characterized by mutations within type VII collagen leading – in most cases – to premature termination of the peptide chain or disturbance of the triple helical structure 12. The acquired form, epidermolysis bullosa acquisita (EBA), is characterized by a loss of tolerance against type VII collagen and serves as a model system for autoimmune skin blistering diseases 1, 13, 14, 15. Several pathogenic epitopes on type VII collagen have been identified in EBA and mouse models have been established featuring certain aspects of the human disease 16. These mouse models are very well characterized in terms of genetic factors leading to skin blistering 17, and also, data on metabolic changes due to the disease have been published 18. Autoantibodies against the vWFA2 domain are pathogenic in an immunization induced mouse model, and approximately 30% of sera from EBA patients recognize the vWFA2 domain 19. Despite the importance of type VII collagen and the multitude of clinical data on type VII collagen related diseases, there is a basic knowledge gap on structural information. Here, we provide first high‐resolution structural and dynamical data for the vWFA2 domain by combining X‐ray crystallography and NMR spectroscopy. Since the structural data showed that the RGD motif is accessible on the protein surface, the interaction of vWFA2 with cellular receptors was investigated in cell adhesion assays. Binding to cells was still observable after mutating the RGD motif; therefore, additional amino acids were mutated based on the protein structure. This finally led to the identification of two interactions that have so far not been attributed to the vWFA2 domain: integrin binding via the RGD motif and binding to laminin‐332. It seems that the vWFA2 domain has additional important functions besides the well‐established collagen binding.

## Materials and methods

### Expression and purification of wild‐type and mutant vWFA2

Unlabelled and ^15^N‐labelled samples of the vWFA2 subdomain (amino acids 1048–1238) of murine type VII collagen have been expressed and purified as described previously 20. In brief, vWFA2 was expressed as fusion protein with an N‐terminal chitin binding tag and a self‐cleaving intein. After cell lysis, the cleared cell lysate was loaded onto a chitin column and self‐cleavage of the intein was induced by a temperature and pH change. After incubation overnight, vWFA2 was eluted. Fractions containing protein were pooled, and buffer was exchanged by ultrafiltration (Amicon Centrifugal filters, Merck Millipore, Carrigtwohill, Ireland; 10 kD MWCO). Mutants of vWFA2 for investigating cell adhesion have been obtained by site‐directed mutagenesis as described in Ref. 11 and purified as wild‐type vWFA2. Initially, the RGD motif of vWFA2 was inversed (R1171D D1173R). Since this mutant (termed DGR mutant) was still positive in the cell attachment assay, additional mutations were introduced: D1209R, D1218R, D1229R, R1120Q K1121R, R1079E R1120Q K1121R, R1120Q K1121R R1225D, R1120Q K1121R D1218R. These amino acids have been selected according to the crystal structure, that is surface accessibility and charges (which are inverted in mutant proteins). Further mutations with an intact RGD motif that has been investigated are as follows: R1120Q K1121R, D1218R and R1225D.

Characterization of vWFA2 laminin‐332 interaction was also carried out with a vWFA2 lacking any tag residues (untagged vWFA2). This protein construct was isolated from cell lysate by a different strategy as described previously 3: after an ammonium sulphate precipitation, a cation exchange chromatography was performed followed by a size exclusion chromatography.

### CD spectroscopy

CD spectra were recorded at 25 °C and 37 °C on a Jasco J‐715 CD photometer (Jasco Corporation, Tokyo, Japan). After subtraction of the blank, molar ellipticities were calculated. For investigating thermal stability of vWFA2, the ellipticity at 220 nm was followed between 25 °C and 90 °C for wild‐type vWFA2 and 5–90 °C for vWFA2 D1218R with a heating rate of 1 K·min^−1^. Protein concentrations were about 10–30 µm in 10 mm sodium phosphate buffer pH 7.4. Melting curves were smoothed by using the Savitzky‐Golay algorithm of the photometer's software.

### Crystallization and structure solution of vWFA2

The vWFA2 subdomain was crystallized at 10 mg·mL^−1^ in 10 mm sodium phosphate buffer pH 7.4 with sitting drop vapour diffusion method in 1.8 m magnesium sulphate, 0.1 m sodium acetate pH 4.6. The crystals were cryoprotected by adding 20% (v/v) glycerol (final) to the crystallization solution. Diffraction of a small crystal (20 × 80 µm) was collected at the ID29 at the ESRF, France 21. Data were integrated using the program xds 22 and further processed using pointless/aimless/ctruncate 23, 24, 25. The phases were solved by molecular replacement with edited PDB entry http://www.rcsb.org/pdb/search/structidSearch.do?structureId=4IGI 26 as a search model using phaser 27. The initial structure was rebuilt with arp/warp 28, 29 and further refined using iterative cycles of coot 30 and phenix.refine 31. Secondary structures were assigned using dssp 32, 33. Figures were generated with pymol (v.1.7 Schrödinger, LLC, New York, NY, USA). Coordinates of vWFA2 have been deposited in the PDB database under the accession number http://www.rcsb.org/pdb/search/structidSearch.do?structureId=6S4C.

### Determination of backbone dynamics and residue dipolar coupling measurements

NMR experiments were performed on a Bruker Avance 500 NMR spectrometer (Bruker, Rheinstetten, Germany) at 298 K (25 °C). The spectrometer was equipped with a CPTCI probe head. NMR samples of vWFA2 contained 10 mm sodium phosphate buffer pH 7.4, 10% (v/v) D_2_O and TSP‐*d*_4_ as internal standard for referencing. The processing of the spectra was done with Topspin 3.1 (Bruker).

Dynamical behaviour of the protein backbone of vWFA2 was investigated by measuring heteronuclear NOEs, *R*_1_ (longitudinal) and *R*_2_ (transversal) relaxation rates. This provides information on the pico‐ to nanosecond timescale 34. Determination of ^1^H‐^15^N heteronuclear NOEs, *R*_1_ and *R*_2_ relaxation rates was done using standard pulse sequences from Bruker. The recovery delay for measurement of *R*_1_ was set to: 10 ms, 50 ms, 70 ms, 0.1 s, 0.2 s, 0.3s, 0.5s, 0.7 s, 1 s, 1.5 s, 3 s and 5 s. For determination of ^15^N‐*T*_2_ relaxation times, the following delay times have been used: 17.0, 33.9, 67.8, 84.8, 101.8, 118.7, 135.7, 152.6, 169.6, 203.5 and 237.4 ms. For measuring ^1^H‐^15^N heteronuclear NOEs, a proton saturation time of 3.7 s was used. Evaluation of the data was done with ccpn 35 by using the published backbone assignment of vWFA2 (BMRB accession number: 17549) 20.

NH‐residual dipolar couplings were measured using the following alignment media: PEG–hexanol [final concentration 4.2% (w/v) PEG] and PEG–hexanol with the positive charged detergent CTAB (1 : 30 CTAB : PEG–hexanol) 36. The pH of the alignment media was adjusted to 7.3–7.4. Alignment was confirmed by measuring the splitting of the deuterium signal of HDO. Coupling constants have been determined with the IPAP‐HSQC experiment 37 using the Bruker pulse sequence (*hsqcf3gpiaphsiwg*). Assignment of the resonances in the IPAP‐HSQC spectra was done with CCPN 35 based on the published backbone assignment of vWFA2 (BMRB accession number: 17549) 20. Determination of the alignment tensor and correlation of experimentally determined residue dipolar couplings (RDCs) with back‐calculated RDC values derived from the crystal structure of vWFA2 was done with pales 38, 39 using singular value decomposition. RDC and dynamical data have been submitted to the BMRB under the entry 50020 (http://www.bmrb.wisc.edu/data_library/summary/index.php?bmrbId=50020).

### Cell adhesion assays

Due to the high conservation of the amino acids in human and murine vWFA2 surrounding the RGD motif, we felt safe using murine vWFA2 in combination with human cell lines. Attachment of human cells to vWFA2‐coated surfaces has been performed using the keratinocyte‐like HaCaT cell line 40 (DKFZ Heidelberg) and unmodified human skin fibroblasts (Coriell Institute for Medical Research, GM21808). The proliferating fibroblasts were at a cumulative population doubling level between 20 and 30. A growth curve of this cell line can be found in Ref. 41. HaCaT cells were grown in CnT‐07 medium (CellNTEC) and the fibroblasts in a 1 : 1 mixture of Ham’s F12 Medium/DMEM supplemented with 2 mm l‐glutamine and 15% fetal bovine serum (not heat inactivated) (Gibco, Life Technologies Corporation, Grand Island, NY, USA). The cell adhesion assay has been performed as described in Ref. 42. In brief, after coating 96‐well cell culture plates (Sarstedt AG & Co. KG, Nürnbrecht, Germany) with protein (three wells per concentration), blocking with BSA and washing with PBS 3–5 × 10^5^ cells have been added in DMEM medium (without FBS). After incubation (37 °C, atmosphere with 5 v/v % CO_2_), nonattached cells were removed by washing with PBS. Adherent cells were fixated with a solution of glutaraldehyde, stained with acridine orange and washed. After lysis, absorption was measured at 490 nm and the mean value and error was calculated. In each cell adhesion assay, fibronectin was included as positive control. When investigating mutations, divalent metal ions or antibodies, also a dilution series of wild‐type vWFA2 was included. Cell adhesion assays have been performed at least in triplicate. The effect of divalent metal ions was investigated in the presence of 2 mm EDTA, 5 mm MgSO_4_ and 5 mm MnSO_4_, respectively. The impact of antibodies on cell adhesion was investigated at 5 µg·mL^−1^ for polyclonal anti‐laminin‐332 antibody (Abcam plc, Cambridge, UK), anti‐β1 integrin (P5D2 abcam, AIIB2 Merck Millipore, Merck KGaA, Darmstadt, Germany) and anti‐αV antibody (272‐17E6; abcam) at least in duplicate measurements.

### SPR measurements

The determination of binding affinities has been done with a BIAcore 3000 (Biacore, Uppsala, Sweden) at 25 °C. Recombinant human laminin‐332 (BioLamina AB, Stockholm, Sweden) was immobilized on a CM5 chip (GE Healthcare Bio‐Sciences AB, Uppsala, Sweden) according to the manufacturer’s protocol to yield a final change of approx. 5000 resonance units (RU) relating to maximal 150 RU assuming a monovalent interaction of vWFA2 and laminin‐332. Running buffer was 10 mm sodium phosphate pH 7.4 150 mm NaCl. For the investigation of the effect of divalent metal ion, MgCl_2_ was added to a final concentration of 2.5 mm to the buffer. About 11 and 14 concentrations (ranging between 2.5 and 420 µm), respectively, of vWFA2 protein were used for the determination of binding isotherms. Data analysis was done with BIAevaluation 4.1 (Biacore).

## Results

As major component of anchoring fibrils, type VII collagen is essential for skin stability by establishing interactions with proteins of the extracellular matrix. The skin blistering autoimmune disease EBA is a model system for investigating triggers leading to the breakdown of immunotolerance against type VII collagen. Although much information on clinical and immunological data is available for autoimmunity against type VII collagen, high‐resolution structural data and information on interactions with other proteins that would improve our understanding of EBA pathogenesis are still missing. Here, we present the first structural and dynamical data at an atomic level for type VII collagen, which finally lead to the identification of two binding sites at vWFA2 domain. These interactions have not been reported so far for the vWFA2 domain.

### Crystallization and measurement of RDC of murine vWFA2

**Figure 2:**
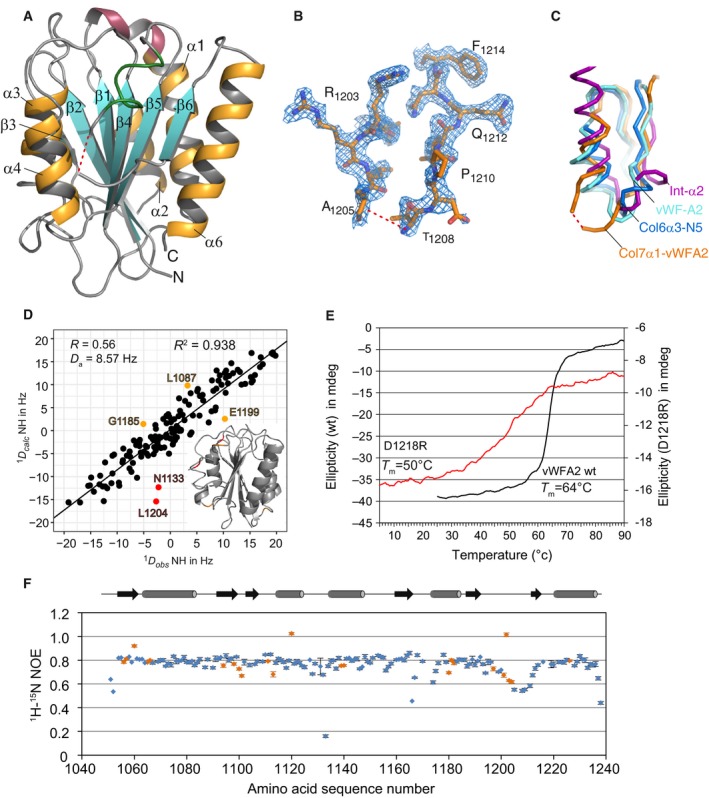
Crystal structure of vWFA2 of type VII collagen. (A) Cartoon diagram of the vWFA2 subdomain of type VII collagen. β strands and α helices of the vWFA fold are labelled β1–β6 and α1–α6. α5 is not detected as an α‐helix by DSSP and is shown as a green ribbon. α1 starts with a short helical turn and a 3_10_ helix indicated in red. Amino acid residues P1206 and G1207 are not visible and are indicated by a dashed line. (B) Electron density around the undefined amino acids P1206 and G1207 contoured at 1σ. (C) Comparison of different loop structures and their adjacent α5 helix (vWF‐A2 domain (light blue; http://www.rcsb.org/pdb/search/structidSearch.do?structureId=3ZQK), the I domain of integrin α2 (purple; http://www.rcsb.org/pdb/search/structidSearch.do?structureId=4BJ3) and the type VI collagen α3 N5 domain (dark blue; http://www.rcsb.org/pdb/search/structidSearch.do?structureId=4IGI). (D) 145 RDCs measured from a PEG–hexanol:CTAB aligned sample correlate well with the theoretical values back‐calculated from the crystal structure (*R* ^2^ = 0.938; *Q* = 0.347, RMSD = 3.11 Hz). Rhombicities (R) of the alignment tensor and the magnitudes (Da) of the alignment tensors are given as inserts. Orange and red values deviate more than 2*RMSD and 3*RMSD from the back‐calculated values, respectively. The following residues are labelled in orange: G1185, L1087 and E1199; and in red: L1204 and N1133. (E) Analysis of thermal stability with CD spectroscopy shows that thermal unfolding of wild‐type vWFA2 starts at 55 °C. The sigmoid melting curve has an inflection point of about 64 °C for wild‐type vWFA2 and 50 °C for the vWFA2 D1218R mutant. (F) Determination of heteronuclear ^1^H‐^15^N NOEs points towards increased flexibility for N1133 and residues around T1208. Residues marked in orange are overlapping in the spectra, which can lead to unreasonable or unreliable values.

We have determined the crystal structure of the vWFA2 subdomain of murine type VII collagen at a resolution of 2.0 Å (Fig. 2A–C). The model was refined to satisfactory statistical values of *R*_work_/*R*_free_ of 0.18/ 0.21 (Table 1). The asymmetric unit contained one molecule spanning from G1052 to A1238. The first nine residues of the vWFA2 construct used in this study, that is five residues belonging to the multiple cloning site and the four N‐terminal residues of vWFA2, are not defined in the crystal structure. Interestingly, this includes the N‐terminal cysteine; consequently, the existing disulphide bridge 20 (see also Fig. S1) is not visible in the electron density map. The overall structure is well defined, with an exception of a small loop stretching from R1203 to I1211. Here, density could be seen, but no satisfactory fit could be achieved (Fig. 2B). Curiously, the crystal contacts are often formed indirectly and not via protein–protein interactions. One crystal contact is formed by a metal‐complexed EDTA molecule. The metal is located on a twofold axis, and the carboxyl groups of EDTA form salt bridges to R1120 of two symmetry mates. Another crystal contact is formed by an inorganic ion (either sulphate or phosphate) sitting on a threefold axis and connecting R1123 of three symmetry‐related molecules. Interestingly, two side‐chain conformations can be observed for K1121. This residue has been shown previously to be crucial for binding type I collagen 11.

**Table 1:** Data collection and refinement statistics for the type VII collagen subdomain vWFA2

	vWFA2 domain of Col7
Data collection	
Beamline	ID29/ESRF
Wavelength (Å)	0.9763
Space group	R32:h (No. 155)
Cell dimensions	
*a*, *b*, *c* (Å)	123.0, 123.0, 63.7
α, β, γ (°)	90.0, 90.0, 120.0
Resolution (Å)	35.5–2.0 (2.05–2.00)^a^
*R*_Sym_	0.139 (0.709)
*R*_meas_	0.157 (0.802)
CC_1/2_	0.995 (0.477)
*I*/σ*I*	6.9 (2.1)
Completeness (%)	99.8 (99.9)
Multiplicity	4.4 (4.6)
Refinement	
Resolution (Å)	35.5–2.0
No. of reflections (test set)	12573 (626)
*R*_work_/*R*_free_ (%)	17.6/20.6
No. atoms	1572
Protein	1415
Ligand/ion	17
Water	140
B‐factors	26.8
Protein	26.2
Ligand/ion	28.2
Water	32.7
R.M.S deviations	
Bond lengths (Å)	0.003
Bond angles (°)	0.76

^a^ Highest resolution shell is shown in parentheses.

The α5 helix is not recognized as an α‐helix by DSSP although it has α‐helical character (Fig. 2A, green ‘loop’ region). The α‐helical conformation for amino acids in this region is supported by NMR chemical shift data that have been determined in an earlier study 20. The α1 helix is directly preceded by a helical turn and a short 3_10_ helix formed by residues 18–22 (Fig. 2A, red helix).

Residue dipolar coupling measurements have been performed to relate the crystal structure with the situation in solution. Alignment of vWFA2 in different alignment media varies as seen by different values measured in a CTAB:PEG–hexanol aligned sample and a PEG–hexanol aligned sample. The measured RDCs are in accordance with the crystal structure. RDCs of backbone amides (NH‐RDC) measured in solution fit well to the crystal structure with sufficient high *R* ^2^ values (Fig. 2D, Fig. S3). The *R* ^2^ of the PEG–hexanol aligned sample is 0.919 (RMSD 1.96 Hz, 139 RDC values used), and for the CTAB:PEG–hexanol aligned sample *R* ^2^ is 0.938 (RMSD: 3.11 Hz, 145 RDC values used) with quality factors (*Q*‐factor 43) of 0.347 (CTAB:PEG–hexanol aligned sample) and 0.392 (PEG–hexanol aligned sample). Residues that show the largest deviation of the back‐calculated RDCs from the measured RDCs are D1114, S1131, V1166, E1199, L1204, A1205 and A1233 for the PEG–hexanol aligned sample and L1087, N1133, G1185, E1199 and L1204 for the CTAB:PEG–hexanol aligned sample. In case of the latter alignment medium, all these residues are within loops, whereas for the PEG–hexanol alignment medium all residues except V1166 and A1233 are within loops. Excluding these residues from the analysis improves the fits substantially, indicating a different conformation in the loop structures (as well as reflecting flexibility) in solution state than in the crystal state for these residues. In case of PEG–hexanol, *R* ^2^ increases to 0.958 (132 values used, *Q* = 0.288, RMSD = 1.46 Hz), and for the CTAB:PEG–hexanol aligned sample, *R* ^2^ increases to 0.957 (140 values used, *Q* = 0.290, RMSD = 2.63 Hz) with satisfactory *Q*‐factors 44, 45, 46. As measured RDCs of amino acids within secondary structure elements are in very good accordance with the crystal structure, we conclude that the crystal structure represents the situation in solution at ambient temperature as well.

### Dynamic behaviour of murine vWFA2

Dynamic properties of the protein backbone can be investigated by measuring *R*_1_ and *R*_2_ relaxation rates. Also, heteronuclear NOEs are very sensitive to backbone flexibility. Analysis of the *R*_1_ and *R*_2_ relaxation rates as well as heteronuclear NOEs shows two regions within vWFA2 that are characterized by increased flexibility as compared to the bulk residues (Fig. 2F, Fig. S4). Besides the terminal residues, N1133, N1219 and residues around A1205 show reduced *R*_2_ relaxation rates. Heteronuclear NOEs are markedly decreased for residues N1133 and T1208. This is indicating local flexibility at the nanoseconds to picoseconds time scale and can be explained by the localization of these residues within loops. This is in line with the crystallographic data where the loop bridging helix α5 with β‐strand β6 is not well defined.

### Thermal stability of vWFA2

CD spectra of vWFA2 at 25 °C and 37 °C are virtually identical with a minimum at 220 nm (Fig. S5). Analysis of the percentage of secondary structure elements using the K2D3 web service 47 resulted in 34% α‐helical and 19% β‐sheet‐like structures, respectively. These values are in very good agreement with the content of α‐helix (36%) and β‐sheet (19%) determined from the crystal structure. The melting temperature of vWFA2 is about 64 °C (Fig. 2E), and thus, vWFA2 is stable also at temperatures considerably above body temperature; that is, at temperatures used for the cell adhesion experiments, vWFA2 is stable and adopts the structure determined by crystallography. Of the mutants generated for identifying the binding sites for cellular receptors (*vide infra*), the vWFA2 R1120Q K1121R D1218R mutant could not be investigated due to low yield after purification. Analysing thermal stability of vWFA2 D1218R shows that this mutant is less stable with a melting temperature of about 50 °C. This demonstrates that D1218 is stabilizing the vWFA2 subdomain and thereby preventing mutations at this site.

### Cell adhesion assays show binding of vWFA2 to integrins and laminin‐332

**Figure 3:**
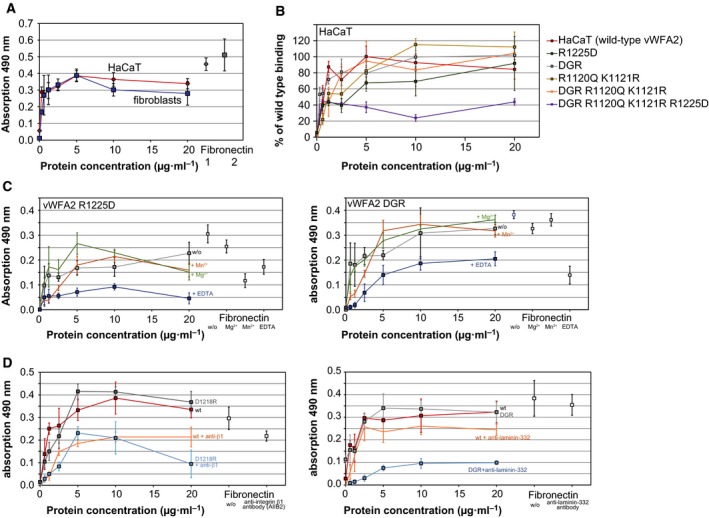
Identification of binding sites for vWFA2. Representative plots of cell adhesion assays are shown. (A) Human skin fibroblasts and the keratinocyte‐like HaCaT cell line show adhesion to vWFA2‐coated surfaces comparable to fibronectin. Mutation of vWFA2 shows decreased binding in case of the DGR R1120Q K1121R R1225D mutant, thereby pointing towards two interactions sites at vWFA2 (B). Data for fibroblasts are shown in Fig. S6. (C) The presence of EDTA shows virtually no binding for the vWFA2 R1225D mutant and reduced binding for the vWFA2 DGR mutant in cell adhesion assays with HaCaT cells. Thus, binding via the RGD motif is depending on divalent metal ions and binding via the binding site at R1225 is at least influenced by the presence of divalent metal ions. Attachment of HaCaT cells is also reduced in the presence of an anti‐integrin β1 antibody for D1218R mutant and anti‐laminin‐332 antibody for the vWFA2 DGR mutant (D). It seems that the anti‐integrin β1 antibody also affects the interaction with laminin‐332 as the wild‐type vWFA2 show reduced binding in the presence of this antibody. This is likely an indirect effect since laminin‐332 is also interacting with integrins. Error bars for cell adhesion plots represent the standard deviation of a triplicate measurement.

vWFA2 contains a potential RGD motif, which is an interaction site for integrins. It was previously shown that the NC1 domain of type VII collagen is interacting with α2β1 integrin 48. The interaction site was located at N‐terminal subdomains by analysing shortened NC1‐fragments; thus, it was concluded that the interaction is RGD independent 48. As seen in the crystal structure, the RGD motif is at the protein surface and the RGD motif is also conserved between different species (see Fig. S7). Thus, it was tested whether vWFA2 can interact with surface proteins of dermal cells. Both keratinocyte‐like HaCaT cells and unmodified human fibroblasts show binding to vWFA2 in a cell attachment assay (Fig. 3A). Mutating the RGD motif to DGR (R1171D D1173R) seemed not to influence cell binding (Fig. 3B, Fig. S6). Since vWFA2 has been shown to be involved in type I collagen, which is produced by fibroblasts, that binding site was also mutated (R1120Q K1121R) to preclude this interaction 11. Since cell attachment seemed not to be affected (Fig. 3B), this led to the assumption that a further interaction site exists. To substantiate this hypothesis, a third mutation was introduced at different positions. Based on the crystal structure, amino acids located on the protein’s surface have been changed. The vWFA2 R1120Q K1121R D1218R mutant could not be tested in the cell attachment assay due to low yield after purification. Therefore, residues nearby this position have been mutated. Of these mutations tested in the cell attachment assay only the mutant containing the following mutations R1225D, mutation of the RGD motif (R1171D, D1173R) and the type I collagen binding site (R1120Q, K1121R) showed reduced binding compared to wild‐type vWFA2. The R1225D mutation alone showed no reduced binding in the cell attachment assay, as the RGD motif is intact.

After establishing the existence of two binding sites on vWFA2 (RGD and R1225) for cell surface receptors, the dependency on divalent metal ions was tested (Fig. 3C) since integrin binding requires divalent metal ions. Binding was not enhanced when Mg^2+^ or Mn^2+^ was added but reduced for each binding site in the presence of EDTA, indicating that the interaction is dependent on divalent metals.

Initial SPR (surface plasmon resonance) experiments pointed towards an interaction of laminin‐332 with vWFA2; hence, a polyclonal anti‐laminin‐332 antibody was tested and reduced binding in the cell adhesion assay was observed for the DGR mutant (Fig. 3D). Investigating different antibodies in their ability to block the interaction of RGD motif with integrins reduced binding was observed with anti‐β1 antibodies but not with anti‐αV antibodies (Fig. 3D, Fig. S6). Also for wild‐type vWFA2, a reduction in cell adhesion was observed in the presence of anti‐β1 antibodies, which is likely an indirect effect since laminin‐332 can also interact with α3β1‐integrin 49.

### Quantification of the laminin‐332 vWFA2 interaction by SPR measurements

**Figure 4:**
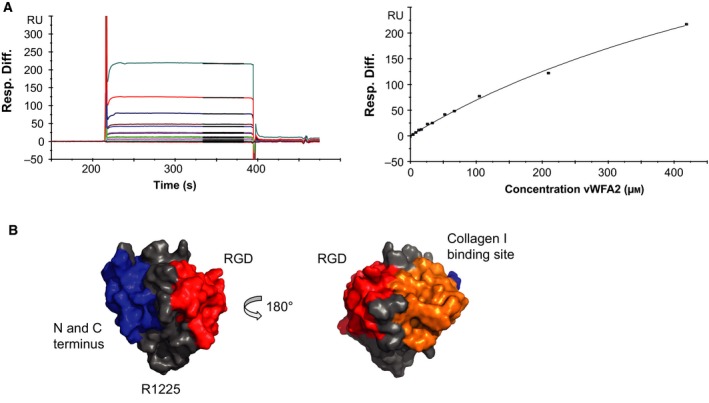
Quantitation of the laminin‐332 interaction. (A) The interaction of vWFA2 and laminin‐332 is confirmed and quantified by SPR analysis yielding a *K*_D_ value of 0.81 mm (*Χ* ^2^ = 8.48). (B) Residues within 10 Å of the N‐/C‐terminus, the RGD motif and the type I collagen binding site are differently coloured to estimate the potential steric demands of these interactions (note that the colour coding is not related to the colours in Fig. 3A–D). This shows that the direct surrounding of R1225 is not involved in any of these interactions, which could reflect the hitherto unknown interaction site.

In order to quantitate the interaction of vWFA2 with laminin‐332, SPR was used (Fig. 4A). Preliminary SPR experiments indicated that the interaction is very weak, and according to literature, the laminin‐332 binding site is located at subdomains FNIII7‐9 of type VII collagen 8, 50. In order to make certain that the observed interaction is not artificial, the *K*_D_ was determined in two independent experiments. Therefore, two different laminin‐332 charges and two different vWFA2 constructs were used: vWFA2 (purified by intein cleavage) showed a *K*_D_ of 0.81 mm (*Χ* ^2^ = 8.48, Fig. 4A) and untagged‐vWFA2 purified by cation exchange chromatography and size exclusion chromatography showed a *K*_D_ of 0.44 mm (*Χ* ^2^ = 0.25). Since it is a low‐affinity interaction and, in the cell attachment assays, residual binding of cells to mutant proteins was still observed, SPR cannot be employed for reliable quantification of the impact of mutations on this interaction. The binding seems not to be dependent on divalent metal ions, since the *K*_D_ of this interaction is 1.1 mm in the presence of Mg^2+^ (*Χ* ^2^ = 5.5).

## Discussion

Autoantibodies against the vWFA2 subdomain of type VII collagen are pathologically relevant in the autoimmune skin blistering disease EBA. This is demonstrated in a mouse model for experimental EBA based on immunization with vWFA2 19. In addition, autoantibodies against vWFA2 are also detected in patients. To improve the understanding of structural consequences caused by autoantibody binding, we structurally characterized the vWA2 domain. Based on the high‐resolution structural data, mutations were introduced leading to the identification of two hitherto unknown binding sites at the vWFA2 domain: laminin‐332 and a β1 integrin.

### Structure of the murine type VII collagen subdomain vWFA2

The structure resembles a typical von Willebrand factor A domain (vWA) fold consisting of a central β‐sheet (β1–β6) surrounded by α‐helices (α1–α4, α6) (Fig. 2A). RDC measurements with NMR spectroscopy prove that at ambient temperature this structure also exists in solution. A search for similar structures using the DALI server 51 identified the vWA domains of type VI collagen 26, αII integrin 52, the von Willebrand factor A2 domain 53 and PTMP‐1 54 as being the most similar structures. In comparison with those structures, our newly solved structure shows a more rigid and straightened α6 helix (Fig. S2). In the crystal structure, it is also seen the side chain of D1218 points towards the N terminus of helix α6 — likely stabilizing the helix. This hypothesis is supported by the fact that the D1218R mutants could only be isolated in low yields and the thermal stability of the D1218R mutant is lower compared to wild‐type vWFA2 (Fig. 2E). The loop connecting the α5 helix and the β6 sheet is not well defined in the structure, indicating a higher degree of flexibility (Fig. 2B). This is in very good agreement with NMR data pointing towards increased backbone flexibility at the nanoseconds to picoseconds time scale in this region (Fig. 2F, Fig. S4). However, the defined parts of this loop differ significantly from loop structures of other vWA domains (Fig. 2C).

The crystal structure shows electrostatic interactions with EDTA and inorganic anions at the type I collagen binding site. As we have previously shown that the interaction of vWFA2 with type I collagen relies on electrostatic interactions 11, it is curious to observe contacts with negative charges in the crystal structure at this position.

### Interactions of the murine type VII collagen subdomain vWFA2

vWFA2 domain has been described as binding site for type I collagen 10, 11. The domain also harbours an RGD motif for which literature claims that it is not involved in integrin binding 48. However, cell adhesion assays with the keratinocyte‐like HaCaT cell line and human normal fibroblasts proved that vWFA2 is binding to cells. Mutation analysis showed that there are two binding sites that allow attachment of cells (Fig. 4B). One binding event is mediated via the RGD motif and is depending on divalent metal ions. Information about the cellular receptor that is involved in this binding was obtained by using different anti‐integrin antibodies – only antibodies against the β1 chain show reduced cell attachment. The second binding site is located at R1225 (see also Fig. S6), and the interaction is inhibited in the presence of anti‐laminin‐332 antibodies. The interaction with laminin‐332 is surprising as earlier reports located this binding site to the FNIII‐like domains 7–9 8, 50. SPR analysis showed that the interaction is of low affinity with a *K*_D_ of about 0.8 mm. Assuming another laminin‐332 binding site in full‐length type VII collagen as described in the literature, this will result in a more affine binding due to multivalency effects. That can explain the difference in affinity of monomeric vWFA2 domain compared to mini‐type VII collagen, which was determined to be 60 nm by Brittingham *et al*. 7. It should also be kept in mind that the vWFA2 domain contains a potential glycosylation, which is missing in the investigated construct due to the expression in *Escherichia coli.* Finally, in this study the interaction of murine vWFA2 and recombinant human laminin‐332 was investigated, whereas the reported values have been determined with purified rat laminin‐332 and human type VII collagen constructs 7. Since native type VII collagen is a homotrimer, that is it contains three vWFA2 domains, thereby increasing the local concentration in the dermal–epidermal junction rendering this interaction more relevant *in vivo*. Due to the high *K*_D_ value, it is not possible to determine the impact of mutations investigated in the cell attachment assay as these mutations showed still binding in the assays. Since laminin‐332 – a component of the basement membrane can bind cellular surface proteins too, the diminished binding in the presence of EDTA can also be an indirect effect, which is supported by SPR showing no enhanced binding of vWFA2 to laminin‐332 in the presence of Mg^2+^.

It is somehow curious that vWFA2 has additional interaction partners besides the well‐established binding to type I collagen 10, 11. It is tentative to speculate that depending on the location of type VII collagen it can exert different functions: when deposited at the dermal–epidermal junction, it mediates stability by binding to type I collagen, but in case of binding to other (cellular) targets, it could activate different signalling pathways, which might be related to wound healing 55.

Future studies should focus on the identification of the integrin ligand. Knowing the integrin partner will allow a more detailed analysis of the accessibility of the vWFA2 binding sites in the skin and exploration of the signalling pathways related to the vWA2 subdomain.

## Concluding remarks

The cause for loss of tolerance against type VII collagen in the development of EBA is unclear. It was currently shown that autoimmunity against vWFA2 causes skin blistering in experimental EBA. These data are now complemented with the first high‐resolution biophysical characterization of the vWFA2 subdomain of type VII collagen. In combination with functional studies, that is identification of two binding sites that have not been so far attributed to the vWFA2 domain, this provides new detailed insights into the function of type VII collagen. It lays the basis for investigating the putative glycosylation site of vWFA2 for a better understanding of type VII collagen function in the extracellular matrix.

## Conflict of interest

The authors declare no conflict of interest.

## Author contributions

UB and KS designed research. JMG and KS wrote the paper. JMG, FF and KS performed the structural analysis; CW and BR did protein isolation and interaction studies.

## Supporting information


**Fig. S1.** In a coomassie stained 15% SDS‐PAGE analysis of murine vWFA2, it shows a different migration behaviour under reducing and non‐reducing conditions, respectively; indicating that a disulphide bridge formed by the two cysteines is present (a). In addition, under non‐reducing conditions also a band at a molecular weight corresponding to a dimer is present. (b) Amino acid sequence of the investigated construct. Residues underlined and in italic font at the N‐terminus belong to the intein tag. The two cysteines forming the disulphide bridge are labelled in orange (residues 1049 and 1235). N1110 is potentially glycosylated.
**Fig. S2.** Overlay of the Col7 vWFA2 domain (orange) with the vWF‐A2 domain (light blue; 3ZQK; RMS: 2.75 Å), the type VI collagen α3 N5 domain (dark blue; 4IGI, RMS: 1.29 Å), the PTMP1‐A1 domain (green, 4CNB_A; RMS: 1.17 Å) and the I domain of integrin α2 (purple; 4BJ3; RMS: 1.66 Å).
**Fig. S3.** (a) 139 RDCs measured from a PEG‐hexanol aligned sample correlate well with the theoretical values back‐calculated from the crystal structure (*R* ^2^ = 0.919, *Q* = 0.392, RMSD = 1.96 Hz). Orange and red values deviate more than 2*RMSD and 3*RMSD from the back‐calculated values, respectively and are plotted on the crystal structure of vWFA2 (b). (c) Secondary structure elements of vWFA2. (d, e) Deviation of measured NH‐RDCs for the individual amino acids (black symbols) compared to the back‐calculated values (gray symbols) according to the crystal structure.
**Fig. S4.** NMR relaxation data of the vWFA2 domain determined by NMR spectroscopy. (a) secondary structure elements of vWFA2. Determination of relaxation rates *R*_1_ (b), *R*_2_ (c), *R*_2_/*R*_1_ (d) and heteronuclear ^1^H‐^15^N NOEs (e) points towards increased flexibility for N1133 and residues around T1208. Residues marked in orange are overlapping in the spectra which can lead to unreasonable values (e.g. as seen for ^1^H‐^15^N NOEs).
**Fig. S5.** CD spectroscopy of vWFA2. CD spectroscopy shows that the overall fold at 25°C and 37°C is identical and the percentages of secondary structure elements are in very good agreement with the crystal structure with 34 % α‐helix and 19 % β‐sheet structures.
**Fig. S6.** Cell adhesion of fibroblasts to vWFA2. The effect of cell adhesion of fibroblasts was investigated by using different vWFA2 mutants. Mutations that show in combination a diminished binding of vWFA2 to fibroblasts due to interference with protein‐protein‐interactions are shown in (a). Mutations, that have also been tested in cell adhesion assay but which do not interfere in binding of vWFA2 since they are distant to the interaction site are shown in (b). (c) Residues within 10 Å of the N‐/C‐terminus, the RGD motif and the type I collagen binding site are differently coloured to estimate the potential steric demands of these interactions. This shows that the direct surrounding of R1225 is not involved in any of these interactions which could reflect the hitherto unknown interaction site. (d) An antibody against β1‐integrin (P5D2) shows a reduction of binding whereas an αV integrin (272‐17E6) antibody does not interfere with cell binding in the cell adhesion assay. Error bars for cell adhesion plots represent the standard deviation of a triplicate measurement.
**Fig. S7.** Differences in surface accessible amino acids near the RGD motif of vWFA2. Surface representation of murine vWFA2 with the amino acids of the RGD motif labelled in red. Residues labelled in gray are identical between human and murine vWFA2 domain whereas amino acids labelled in yellow are different showing that the surrounding of the RGD motif is quite conserved between the two species. Sequence identity between the two molecules is 80%.

## Data Availability

Coordinates and structure factors of vWFA2 have been deposited in the PDB database (accession number http://www.rcsb.org/pdb/search/structidSearch.do?structureId=6S4C). RDC and dynamical data have been deposited at the BMRB (entry‐ID 50020, http://www.bmrb.wisc.edu/data_library/summary/index.php?bmrbId=50020).

## References

[1] Gupta R , Woodley DT and Chen M (2012) Epidermolysis bullosa acquisita. Clin Dermatol 30, 60–69. 22137228 10.1016/j.clindermatol.2011.03.011 PMC3234994

[2] Goletz S , Zillikens D and Schmidt E (2017) Structural proteins of the dermal‐epidermal junction targeted by autoantibodies in pemphigoid diseases. Exp Dermatol 26, 1154–1162. 28887824 10.1111/exd.13446

[3] Wegener H , Paulsen H and Seeger K (2014) The cysteine rich region of type VII collagen is a cystine knot with a new topology. J Biol Chem 289, 4861–4869. 24385431 10.1074/jbc.M113.531327 PMC3931048

[4] Bächinger HP , Morris NP , Lunstrum GP , Keene DR , Rosenbaum LM , Compton LA and Burgeson RE (1990) The relationship of the biophysical and biochemical characteristics of type VII collagen to the function of anchoring fibrils. J Biol Chem 265, 10095–10101. 2112541

[5] Richer BC and Seeger K (2014) The hinge region of type VII collagen is intrinsically disordered. Matrix Biol 36, 77–83. 24810542 10.1016/j.matbio.2014.04.006

[6] Christiano AM , Rosenbaum LM , Chung‐Honet LC , Parente MG , Woodley DT , Pan T‐C , Zhang RZ , Chu M‐L , Burgeson RE and Uitto J (1992) The large non‐collagenous domain (NC‐1) of type VII collagen is amino‐terminal and chimeric. Homology to cartilage matrix protein, the type III domains of fibronectin and the A domains of von Willebrand factor. Hum Mol Genet 1, 475–481. 1307247 10.1093/hmg/1.7.475

[7] Brittingham R , Uitto J and Fertala A (2006) High‐affinity binding of the NC1 domain of collagen VII to laminin 5 and collagen IV. Biochem Biophys Res Commun 343, 692–699. 16563355 10.1016/j.bbrc.2006.03.034

[8] Chen M , Marinkovich MP , Jones JCR , O'Toole EA , Li Y‐Y and Woodley DT (1999) NC1 domain of type VII collagen binds to the [beta]3 chain of laminin 5 via a unique subdomain within the fibronectin‐like repeats. J Invest Dermatol 112, 177–183. 9989793 10.1046/j.1523-1747.1999.00491.x

[9] Chen M , Marinkovich MP , Veis A , Cai X , Rao CN , O'Toole EA and Woodley DT (1997) Interactions of the amino‐terminal noncollagenous (NC1) domain of type VII collagen with extracellular matrix components. J Biol Chem 272, 14516–14522. 9169408 10.1074/jbc.272.23.14516

[10] Villone D , Fritsch A , Koch M , Bruckner‐Tuderman L , Hansen U and Bruckner P (2008) Supramolecular interactions in the dermo‐epidermal junction zone. J Biol Chem 283, 24506–24513. 18599485 10.1074/jbc.M802415200 PMC3259843

[11] Wegener H , Leineweber S and Seeger K (2013) The vWFA2 domain of type VII collagen is responsible for collagen binding. Biochem Biophys Res Commun 430, 449–453. 23237810 10.1016/j.bbrc.2012.11.119

[12] Dang N and Murrell DF (2008) Mutation analysis and characterization of COL7A1 mutations in dystrophic epidermolysis bullosa. Exp Dermatol 17, 553–568. 18558993 10.1111/j.1600-0625.2008.00723.x

[13] Ludwig RJ (2013) Clinical presentation, pathogenesis, diagnosis, and treatment of epidermolysis bullosa acquisita. ISRN Dermatol 2013, 25. 10.1155/2013/812029 PMC3727188 23956869

[14] Chen M , Kim GH , Prakash L and Woodley DT (2012) Epidermolysis bullosa acquisita: autoimmunity to anchoring fibril collagen. Autoimmunity 45, 91–101. 21955050 10.3109/08916934.2011.606450 PMC3411315

[15] Ludwig RJ , Vanhoorelbeke K , Leypoldt F , Kaya Z , Bieber K , McLachlan SM , Komorowski L , Luo J , Cabral‐Marques O , Hammers CM *et al* (2017) Mechanisms of autoantibody‐induced pathology. Front Immunol 8, 603. 28620373 10.3389/fimmu.2017.00603 PMC5449453

[16] Ludwig RJ (2012) Model systems duplicating epidermolysis bullosa acquisita: a methodological review. Autoimmunity 45, 102–110. 21923614 10.3109/08916934.2011.606451

[17] Ludwig RJ , Recke A , Bieber K , Müller S , Marques AC , Banczyk D , Hirose M , Kasperkiewicz M , Ishii N , Schmidt E *et al* (2011) Generation of antibodies of distinct subclasses and specificity is linked to H2s in an active mouse model of epidermolysis bullosa acquisita. J Invest Dermatol 131, 167–176. 20720563 10.1038/jid.2010.248

[18] Schönig S , Recke A , Hirose M , Ludwig R and Seeger K (2013) Metabolite analysis distinguishes between mice with epidermolysis bullosa acquisita and healthy mice. Orphanet J Rare Dis 8, 93. 23800341 10.1186/1750-1172-8-93 PMC3703300

[19] Iwata H , Bieber K , Tiburzy B , Chrobok N , Kalies K , Shimizu A , Leineweber S , Ishiko A , Vorobyev A , Zillikens D *et al* (2013) B cells, dendritic cells, and macrophages are required to induce an autoreactive CD4 helper T cell response in experimental epidermolysis bullosa acquisita. J Immunol 191, 2978–2988. 23960233 10.4049/jimmunol.1300310

[20] Leineweber S , Schönig S and Seeger K (2011) Insight into interactions of the von‐Willebrand‐factor‐A‐like domain 2 with the FNIII‐like domain 9 of collagen VII by NMR and SPR. FEBS Lett 585, 1748–1752. 21570975 10.1016/j.febslet.2011.04.071

[21] de Sanctis D , Beteva A , Caserotto H , Dobias F , Gabadinho J , Giraud T , Gobbo A , Guijarro M , Lentini M , Lavault B *et al* (2012) ID29: a high‐intensity highly automated ESRF beamline for macromolecular crystallography experiments exploiting anomalous scattering. J Synchrotron Radiat 19, 455–461. 22514185 10.1107/S0909049512009715

[22] Kabsch W (2012) XDS. Acta Crystallogr D 66, 125–132. 10.1107/S0907444909047337 PMC2815665 20124692

[23] Evans PR and Murshudov GN (2013) How good are my data and what is the resolution? Acta Crystallogr D 69, 1204–1214. 23793146 10.1107/S0907444913000061 PMC3689523

[24] Evans PR (2011) An introduction to data reduction: space‐group determination, scaling and intensity statistics. Acta Crystallogr D 67, 282–292. 21460446 10.1107/S090744491003982X PMC3069743

[25] Evans P (2005) Scaling and assessment of data quality. Acta Crystallogr D 62, 72–82. 16369096 10.1107/S0907444905036693

[26] Becker AK , Mikolajek H , Paulsson M , Wagener R and Werner JM (2013) A structure of a collagen VI VWA domain displays N and C termini at opposite sides of the protein. Structure 22, 199–208. 24332716 10.1016/j.str.2013.06.028 PMC3919171

[27] McCoy AJ , Grosse‐Kunstleve RW , Adams PD , Winn MD , Storoni LC and Read RJ (2007) Phaser crystallographic software. J Appl Crystallogr 40, 658–674. 19461840 10.1107/S0021889807021206 PMC2483472

[28] Langer G , Cohen SX , Lamzin VS and Perrakis A (2008) Automated macromolecular model building for X‐ray crystallography using ARP/wARP version 7. Nat Protoc 3, 1171–1179. 18600222 10.1038/nprot.2008.91 PMC2582149

[29] Murshudov GN , Skubak P , Lebedev AA , Pannu NS , Steiner RA , Nicholls RA , Winn MD , Long F and Vagin AA (2011) *REFMAC* 5 for the refinement of macromolecular crystal structures. Acta Crystallogr D 67, 355–367. 21460454 10.1107/S0907444911001314 PMC3069751

[30] Emsley P , Lohkamp B , Scott WG and Cowtan K (2010) Features and development of Coot. Acta Crystallogr D 66, 486–501. 20383002 10.1107/S0907444910007493 PMC2852313

[31] Adams PD , Afonine PV , Bunkoczi G , Chen VB , Davis IW , Echols N , Headd JJ , Hung L‐W , Kapral GJ , Grosse‐Kunstleve RW *et al* (2010) *PHENIX* : a comprehensive Python‐based system for macromolecular structure solution. Acta Crystallogr D 66, 213–221. 20124702 10.1107/S0907444909052925 PMC2815670

[32] Kabsch W and Sander C (1983) Dictionary of protein secondary structure: pattern recognition of hydrogen‐bonded and geometrical features. Biopolymers 22, 2577–2637. 6667333 10.1002/bip.360221211

[33] Joosten RP , te Beek TA , Krieger E , Hekkelman ML , Hooft RW , Schneider R , Sander C and Vriend G (2011) A series of PDB related databases for everyday needs. Nucleic Acids Res 39, D411–D419. 21071423 10.1093/nar/gkq1105 PMC3013697

[34] Morin S (2011) A practical guide to protein dynamics from ^15^N spin relaxation in solution. Prog Nucl Magn Reson Spectrosc 59, 245–262. 21920220 10.1016/j.pnmrs.2010.12.003

[35] Vranken WF , Boucher W , Stevens TJ , Fogh RH , Pajon A , Llinas M , Ulrich EL , Markley JL , Ionides J and Laue ED (2005) The CCPN data model for NMR spectroscopy: development of a software pipeline. Proteins 59, 687–696. 15815974 10.1002/prot.20449

[36] Rückert M and Otting G (2000) Alignment of biological macromolecules in novel nonionic liquid crystalline media for NMR experiments. J Am Chem Soc 122, 7793–7797.

[37] Ottiger M , Delaglio F and Bax A (1998) Measurement of J and dipolar couplings from simplified two‐dimensional NMR spectra. J Magn Reson 131, 373–378. 9571116 10.1006/jmre.1998.1361

[38] Zweckstetter M (2008) NMR: prediction of molecular alignment from structure using the PALES software. Nat Protocols 3, 679–690. 18388951 10.1038/nprot.2008.36

[39] Zweckstetter M and Bax A (2000) Prediction of sterically induced alignment in a dilute liquid crystalline phase: Aid to protein structure determination by NMR. J Am Chem Soc 122, 3791–3792.

[40] Boukamp P , Petrussevska RT , Breitkreutz D , Hornung J , Markham A and Fusenig NE (1988) Normal keratinization in a spontaneously immortalized aneuploid human keratinocyte cell line. J Cell Biol 106, 761. 2450098 10.1083/jcb.106.3.761 PMC2115116

[41] Windler C , Gey C and Seeger K (2017) Skin melanocytes and fibroblasts show different changes in choline metabolism during cellular senescence. Mech Ageing Dev 164, 82–90. 28476532 10.1016/j.mad.2017.05.001

[42] Gebauer JM , Keene DR , Olsen BR , Sorokin LM , Paulsson M and Wagener R (2009) Mouse AMACO, a kidney and skin basement membrane associated molecule that mediates RGD‐dependent cell attachment. Matrix Biol 28, 456–462. 19651211 10.1016/j.matbio.2009.07.006

[43] Cornilescu G , Marquardt JL , Ottiger M and Bax A (1998) Validation of protein structure from anisotropic carbonyl chemical shifts in a dilute liquid crystalline phase. J Am Chem Soc 120, 6836–6837.

[44] Bax A and Grishaev A (2005) Weak alignment NMR: a hawk‐eyed view of biomolecular structure. Curr Opin Struct Biol 15, 563–570. 16140525 10.1016/j.sbi.2005.08.006

[45] Mukherjee M , Dutta K , White MA , Cowburn D and Fox RO (2006) NMR solution structure and backbone dynamics of domain III of the E protein of tick‐borne Langat flavivirus suggests a potential site for molecular recognition. Protein Sci 15, 1342–1355. 16731969 10.1110/ps.051844006 PMC2242546

[46] Wang X , Bansal S , Jiang M and Prestegard JH (2008) RDC‐assisted modeling of symmetric protein homo‐oligomers. Protein Sci 17, 899–907. 18436958 10.1110/ps.073395108 PMC2327283

[47] Louis‐Jeune C , Andrade‐Navarro MA and Perez‐Iratxeta C (2012) Prediction of protein secondary structure from circular dichroism using theoretically derived spectra. Proteins 80, 374–381. 22095872 10.1002/prot.23188

[48] Chen M , O'Toole EA , Li Y‐Y and Woodley DT (1999) α2β1 integrin mediates dermal fibroblast attachment to type VII collagen via a 158‐amino‐acid segment of the NC1 domain. Exp Cell Res 249, 231–239. 10366422 10.1006/excr.1999.4473

[49] Künneken K , Pohlentz G , Schmidt‐Hederich A , Odenthal U , Smyth N , Peter‐Katalinic J , Bruckner P and Eble JA (2004) Recombinant human laminin‐5 domains – effects of heterotrimerization, proteolytic processing, and N‐glycosylation on α3β1 integrin binding. J Biol Chem 279, 5184–5193. 14612440 10.1074/jbc.M310424200

[50] Ortiz‐Urda S , Garcia J , Green CL , Chen L , Lin Q , Veitch DP , Sakai LY , Lee H , Marinkovich MP and Khavari PA (2005) Type VII collagen is required for Ras‐driven human epidermal tumorigenesis. Science 307, 1773–1776. 15774758 10.1126/science.1106209

[51] Holm L and Laakso LM (2016) Dali server update. Nucleic Acids Res 44, W351–W355. 27131377 10.1093/nar/gkw357 PMC4987910

[52] Carafoli F , Hamaia SW , Bihan D , Hohenester E and Farndale RW (2013) An activating mutation reveals a second binding node of the integrin α2 I domain to the GFOGER motif in collagens. PLoS ONE 8, e69833. 23922814 10.1371/journal.pone.0069833 PMC3726769

[53] Jakobi AJ , Mashaghi A , Tans SJ and Huizinga EG (2011) Calcium modulates force sensing by the von Willebrand factor A2 domain. Nat Commun 2, 385. 21750539 10.1038/ncomms1385 PMC3144584

[54] Suhre MH , Gertz M , Steegborn C and Scheibel T (2014) Structural and functional features of a collagen‐binding matrix protein from the mussel byssus. Nat Commun 5, 3392. 24569701 10.1038/ncomms4392

[55] Nyström A , Velati D , Mittapalli VR , Fritsch A , Kern JS and Bruckner‐Tuderman L (2013) Collagen VII plays a dual role in wound healing. J Clin Invest 123, 3498–3509. 23867500 10.1172/JCI68127 PMC3726167

