# Supplementary material for: Structural and biophysical characterization of the type VII collagen vWFA2 subdomain leads to identification of two binding sites
Source: FEBS Open Bio. 2020 Mar 14;10(4):580–92. doi: 10.1002/2211-5463.12807 (PMC7137805; doi:10.1002/2211-5463.12807)
Supplement: Supplementary file 1 — Fig. S1. In a coomassie stained 15% SDS‐PAGE analysis of murine vWFA2, it shows a different migration behaviour under reducing and non‐reducing conditions, respectively; indicating that a disulphide bridge formed by the two cysteines is present (a). In addition, under non‐reducing conditions also a band at a molecular weight corresponding to a dimer is present. (b) Amino acid sequence of the investigated construct. Residues underlined and in italic font at the N‐terminus belong to the intein tag. The two cysteines forming the disulphide bridge are labelled in orange (residues 1049 and 1235). N1110 is potentially glycosylated. Fig. S2. Overlay of the Col7 vWFA2 domain (orange) with the vWF‐A2 domain (light blue; 3ZQK; RMS: 2.75 Å), the type VI collagen α3 N5 domain (dark blue; 4IGI, RMS: 1.29 Å), the PTMP1‐A1 domain (green, 4CNB_A; RMS: 1.17 Å) and the I domain of integrin α2 (purple; 4BJ3; RMS: 1.66 Å). Fig. S3. (a) 139 RDCs measured from a PEG‐hexanol aligned sample correlate well with the theoretical values back‐calculated from the crystal structure (R 2 = 0.919, Q = 0.392, RMSD = 1.96 Hz). Orange and red values deviate more than 2*RMSD and 3*RMSD from the back‐calculated values, respectively and are plotted on the crystal structure of vWFA2 (b). (c) Secondary structure elements of vWFA2. (d, e) Deviation of measured NH‐RDCs for the individual amino acids (black symbols) compared to the back‐calculated values (gray symbols) according to the crystal structure. Fig. S4. NMR relaxation data of the vWFA2 domain determined by NMR spectroscopy. (a) secondary structure elements of vWFA2. Determination of relaxation rates R 1 (b), R 2 (c), R 2/R 1 (d) and heteronuclear 1H‐15N NOEs (e) points towards increased flexibility for N1133 and residues around T1208. Residues marked in orange are overlapping in the spectra which can lead to unreasonable values (e.g. as seen for 1H‐15N NOEs). Fig. S5. CD spectroscopy of vWFA2. CD spectroscopy shows that the overall fold at 25°C and [file FEB4-10-580-s001.pdf]

Structural and biophysical characterization of the type VII collagen vWFA2 subdomain leads to identification of two binding sites.

Jan M. Gebauer[1,3], Florian Flachsenberg[2,3,4], Cordula Windler[2], Barbara Richer[2], Ulrich Baumann[1], and Karsten Seeger[2]*

[1] Institute of Biochemistry, University of Cologne, Otto-Fischer-Str. 12-14, 50674 Köln, Germany

[2] Institute of Chemistry and Metabolomics, University of Lübeck, Ratzeburger Allee 160, 23562 Lübeck, Germany

[3] JMG and FF contributed equally

[4] current address: Universität Hamburg, ZBH - Center for Bioinformatics, Bundesstr. 43, 20146 Hamburg, Germany

* To whom correspondence may be addressed:

Karsten Seeger, Institute of Chemistry and Metabolomics, University of Lübeck, Ratzeburger Allee 160, 23562 Lübeck, Germany

email: karsten.seeger@chemie.uni-luebeck.de

telephone: 0049-451-3101-3334

fax: 0049-451-3101-3304

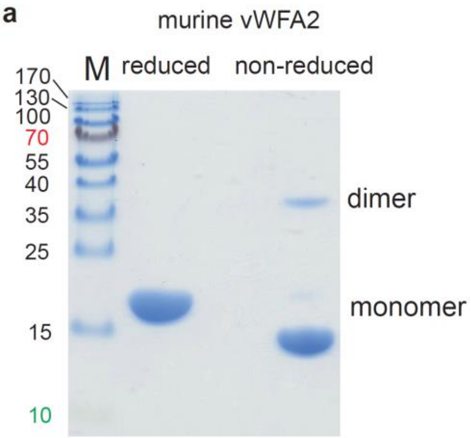

b

1050 1060 1070 1080
*GRAMG*ACS HGPVDVVFLL HATRDNAHNA EAVRRVLERL

1090 1100 1110 1120
VSALGPLGPQ AAQVGLLTYS HRPSPLFPLN SSHDLGIILR

1130 1140 1150 1160
KIRDIPYVDP SGNNLGTAVT TAHRYLLASN APGRRQQVPG

1170 1180 1190 1200
VMVLLVDEPL RGDILSPIRE AQTSGLKVMA LSLVGADPEQ

1210 1220 1230
LRRLAPGTDP IQNFFAVDNG PGLDRAVSDL AVALCQAA

Figure S1: In a coomassie stained 15% SDS-PAGE analysis of murine vWFA2, it shows a different migration behaviour under reducing and non-reducing conditions, respectively; indicating that a disulphide bridge formed by the two cysteines is present (**a**). In addition, under non-reducing conditions also a band at a molecular weight corresponding to a dimer is present. (**b**) Amino acid sequence of the investigated construct. Residues underlined and in italic font at the N-terminus belong to the intein tag. The two cysteines forming the disulphide bridge are labelled in orange (residues 1049 and 1235). N1110 is potentially glycosylated.

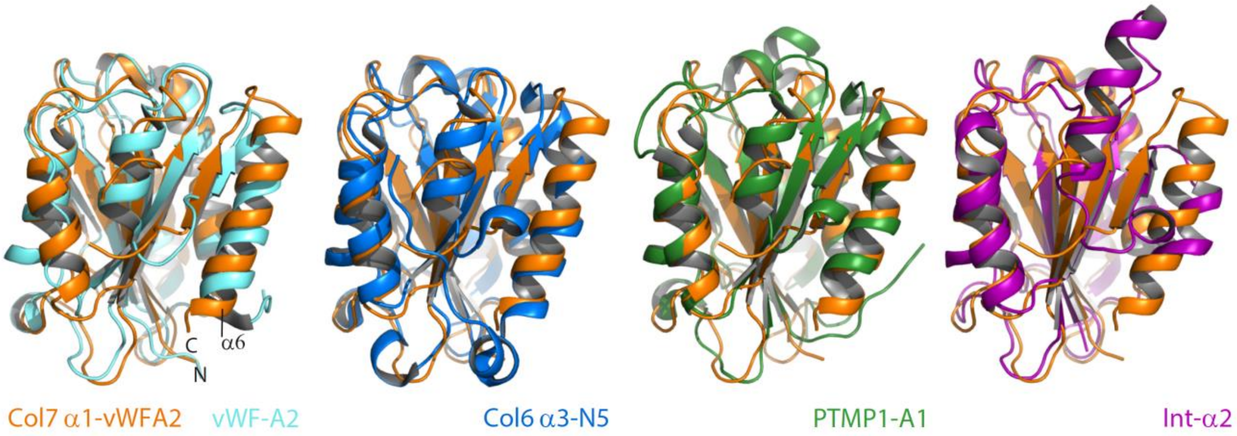

Figure S2: Overlay of the Col7 vWFA2 domain (orange) with the vWF-A2 domain (light blue; 3ZQK; RMS: 2.75 Å), the type VI collagen α3 N5 domain (dark blue; 4IGI, RMS: 1.29 Å), the PTMP1-A1 domain (green, 4CNB_A; RMS: 1.17 Å) and the I domain of integrin α2 (purple; 4BJ3; RMS: 1.66 Å).

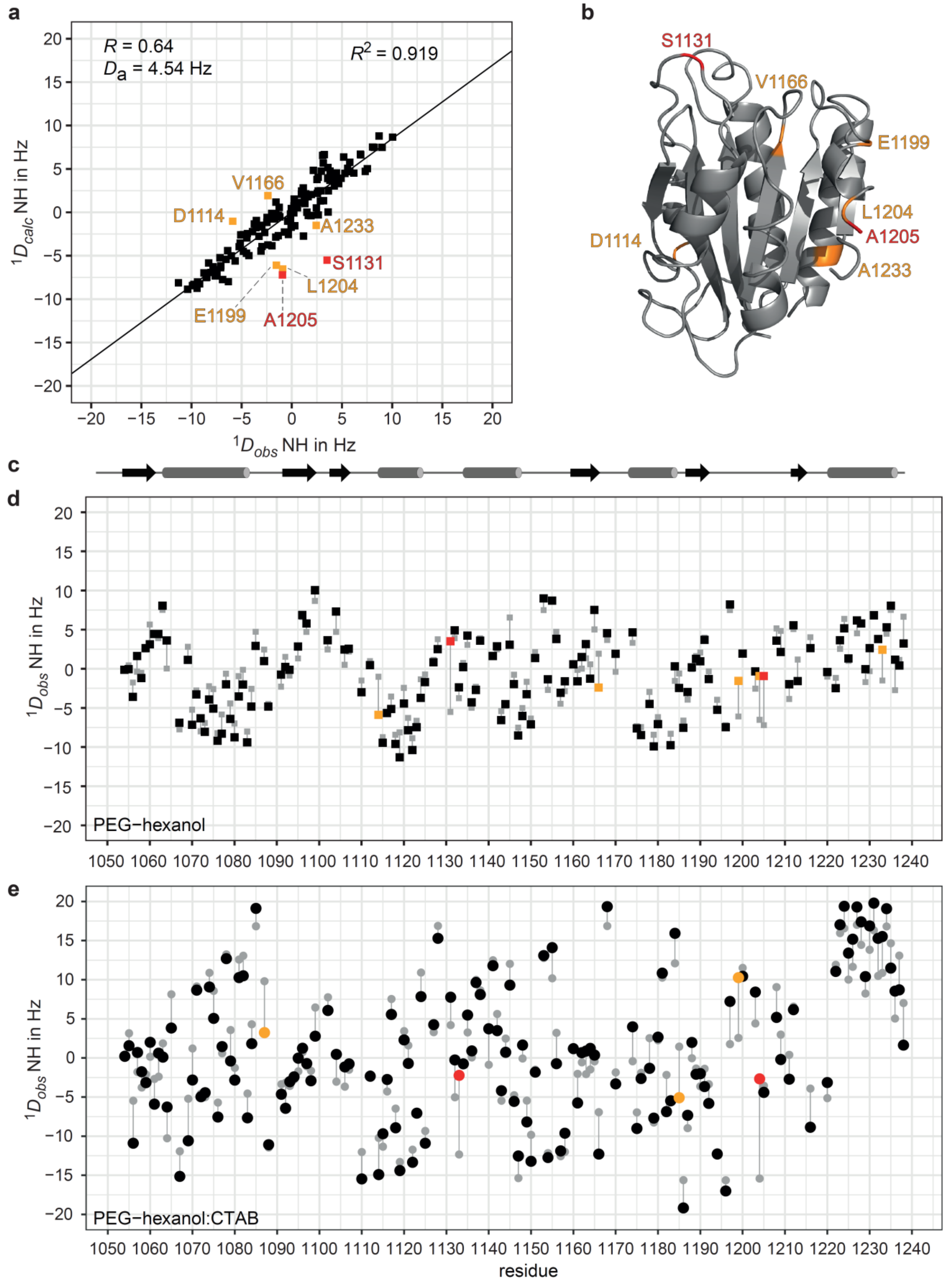

Figure S3: (**a**) 139 RDCs measured from a PEG-hexanol aligned sample correlate well with the theoretical values back-calculated from the crystal structure (R^2 = 0.919, Q = 0.392, RMSD = 1.96 Hz). Orange and red values deviate more than 2*RMSD and 3*RMSD from the back-calculated values, respectively and are plotted on the crystal structure of vWFA2 (**b**). (**c**) Secondary structure elements of vWFA2. (**d, e**) Deviation of measured NH-RDCs for the individual amino acids (black symbols) compared to the back-calculated values (gray symbols) according to the crystal structure.

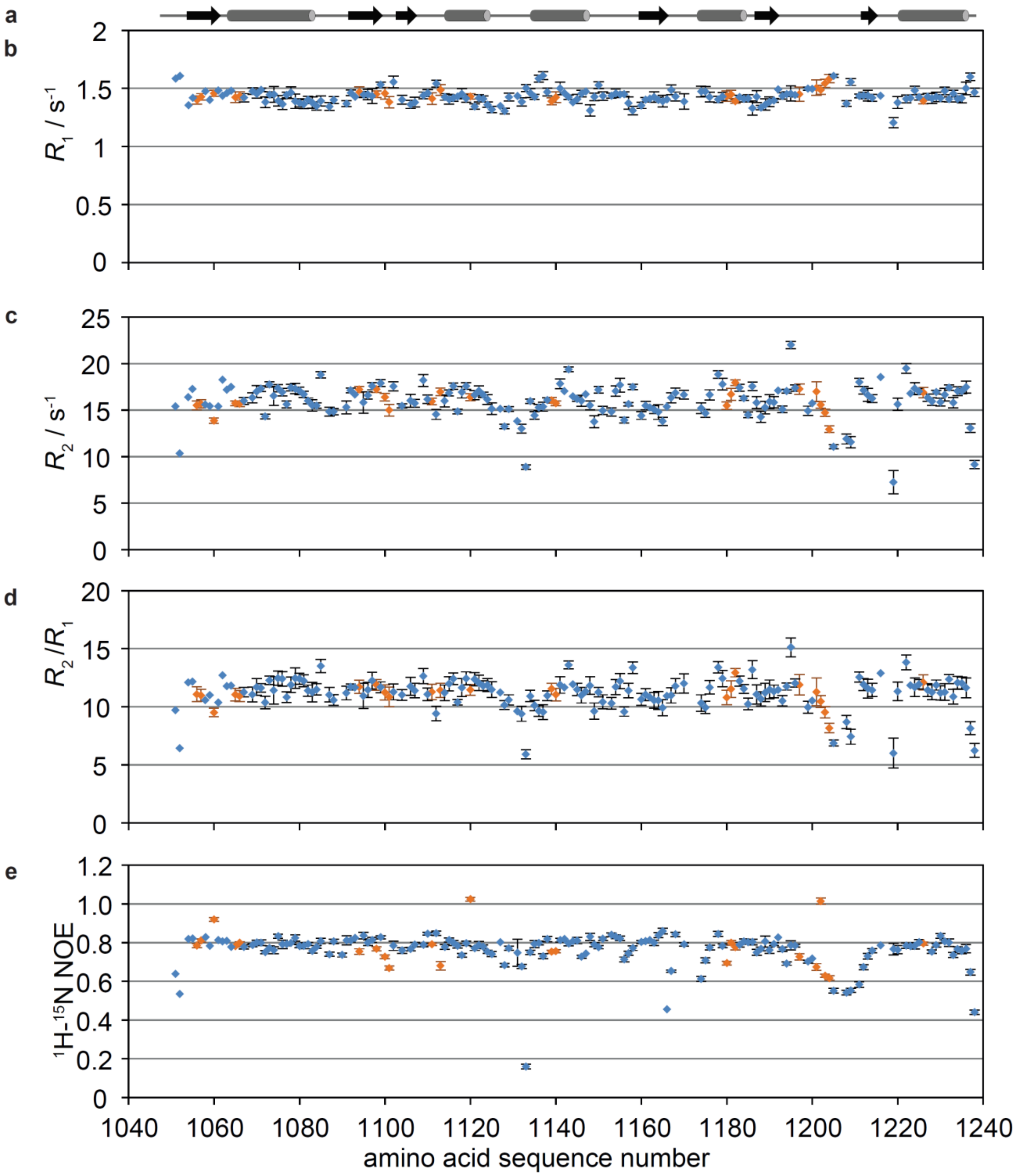

Figure S4: NMR relaxation data of the vWFA2 domain determined by NMR spectroscopy. (**a**) secondary structure elements of vWFA2. Determination of relaxation rates R_1 (**b**), R_2 (**c**), R_2/R_1 (**d**) and heteronuclear ^{1}H-^{15}N NOEs (**e**) points towards increased flexibility for N1133 and residues around T1208. Residues marked in orange are overlapping in the spectra which can lead to unreasonable values (e.g. as seen for ^{1}H-^{15}N NOEs).

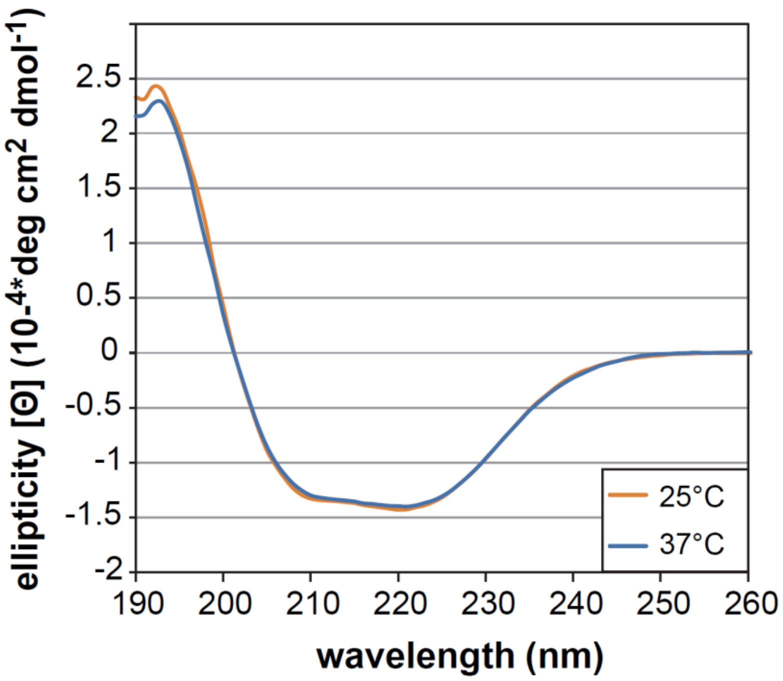

Figure S5 CD spectroscopy of vWFA2. CD spectroscopy shows that the overall fold at 25°C and 37°C is identical and the percentages of secondary structure elements are in very good agreement with the crystal structure with 34 % α-helix and 19 % β-sheet structures.

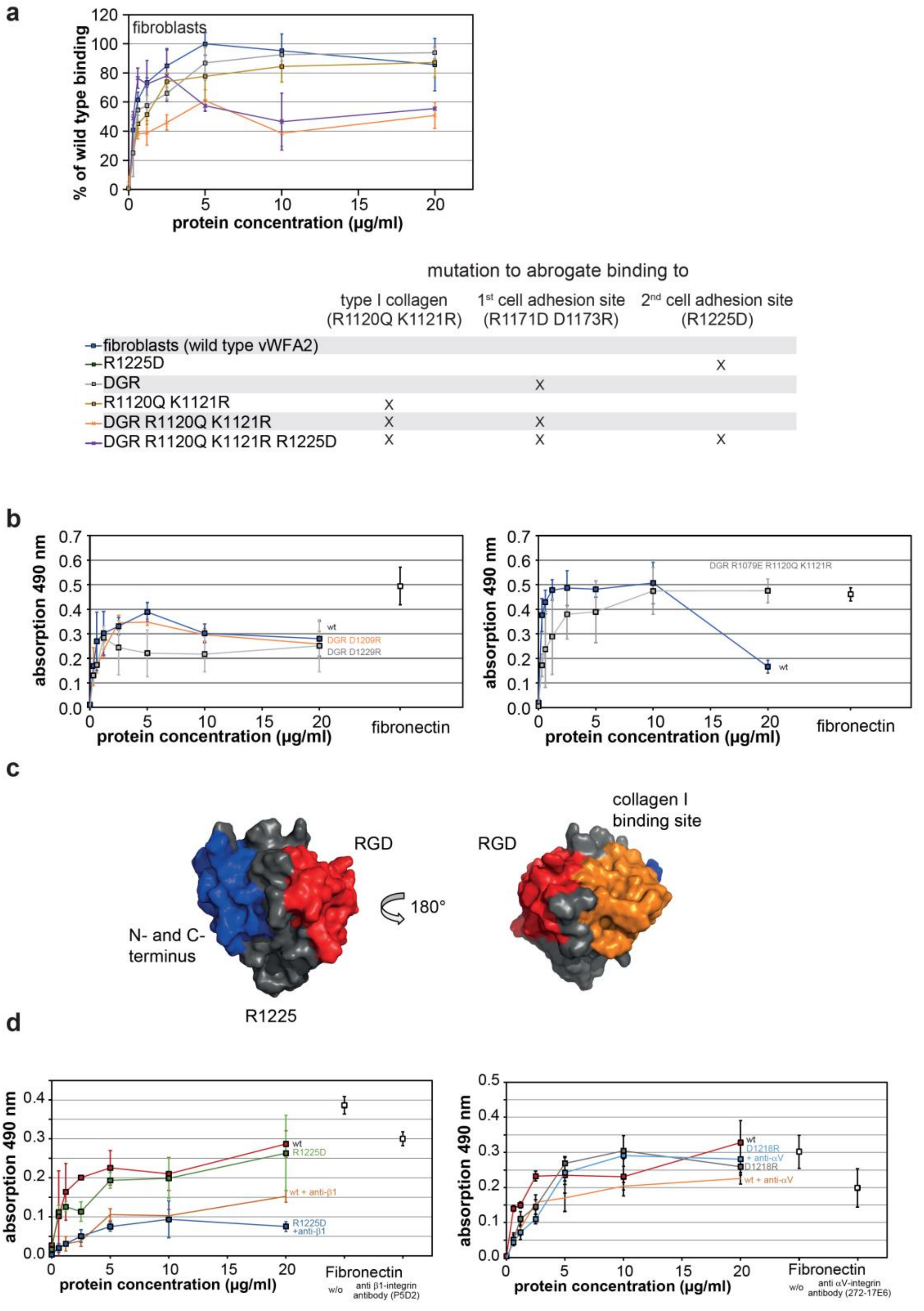

Figure S6 Cell adhesion of fibroblasts to vWFA2. The effect of cell adhesion of fibroblasts was investigated by using different vWFA2 mutants. Mutations that show in combination a diminished binding of vWFA2 to fibroblasts due to interference with protein-protein-interactions are shown in (**a**). Mutations, that have also been tested in cell adhesion assay but which do not interfere in binding of vWFA2 since they are distant to the interaction site are shown in (**b**). (**c**) Residues within 10 Å of the N-/C-terminus, the RGD motif and the type I collagen binding site are differently coloured to estimate the potential steric demands of these interactions. This shows that the direct surrounding of R1225 is not involved in any of these interactions which could reflect the hitherto unknown interaction site. (**d**) An antibody against β1-integrin (P5D2) shows a reduction of binding whereas an αV integrin (272-17E6) antibody does not interfere with cell binding in the cell adhesion assay. Error bars for cell adhesion plots represent the standard deviation of a triplicate measurement.

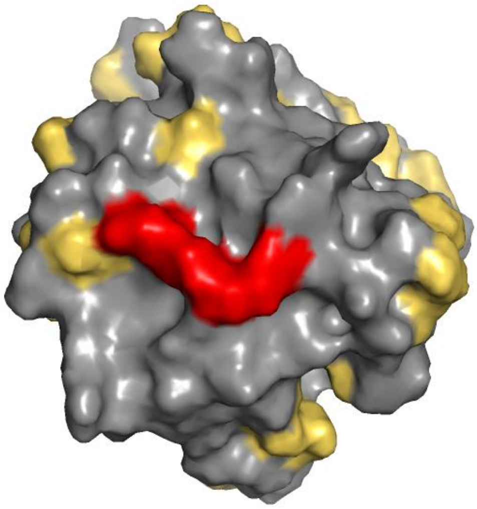

Figure S7 Differences in surface accessible amino acids near the RGD motif of vWFA2. Surface representation of murine vWFA2 with the amino acids of the RGD motif labelled in red. Residues labelled in gray are identical between human and murine vWFA2 domain whereas amino acids labelled in yellow are different showing that the surrounding of the RGD motif is quite conserved between the two species. Sequence identity between the two molecules is 80%.
